# Workplace Violence Against Doctors in India: A Traditional Review

**DOI:** 10.7759/cureus.8706

**Published:** 2020-06-20

**Authors:** Sankuru Santosh K Dora, Humera Batool, Rifath I Nishu, Pousettef Hamid

**Affiliations:** 1 Anesthesia, California Institute of Behavioral Neurosciences and Psychology, Fairfield, USA; 2 Research, California Institute of Behavorial Neurosciences and Psychology, Fairfield, USA; 3 Internal Medicine, California Institute of Behavioral Neurosciences and Psychology, Fairfield, USA; 4 Neurology, California Institute of Behavioral Neurosciences and Psychology, Fairfield, USA

**Keywords:** workplace violence, doctors, india

## Abstract

Workplace violence against doctors is not new, but in recent times, it has grown up in epidemic proportions. Doctors are more worried about their safety and life in the workplace. Meager government spending on healthcare associated with the poor socioeconomic status of the patient and the ever-rising cost of treatment had worsened the situation in present times. The article aims to address this critical issue and try to find possible ways to prevent it.

## Introduction and background

According to the WHO framework Guidelines (2002), “Workplace violence is defined as the situations where staffs are ill-treated, intimidated or attacked in conditions linked to their workplace, including commuting to and from the workplace, involving an explicit or implicit challenge to their safety, well-being or health” [1]. Compared to all other workers, workplace violence seen in healthcare workers is four times higher and hence requires a longer time away from work [2]. It can be physical violence or psychological violence or a combination of both. It can be in any form like assault, abuse, bullying, mobbing, harassment either sexual or racial or psychological, threat, etc. [1].

**Figure 1:**
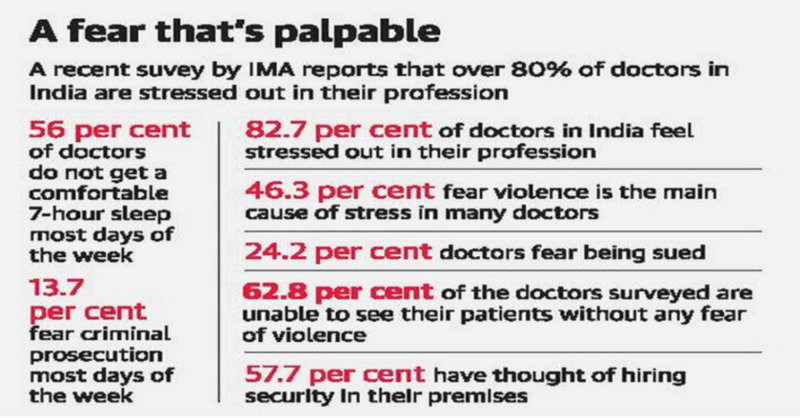
Violence and stress among Indian Doctors (Indian Medical Association) (Retrieved from https://www.thehindu.com/sci-tech/health/majority-of-doctors-in-india-fear-violence-says-ima-survey/article19198919.ece.)

Workplace violence against doctors is a global phenomenon. It has neither any region nor religion. It is prevalent not only in India, Pakistan, Bangladesh but also in developed countries like the USA, UK, and China. It is spread across the continents from Asia to Australia, Europe to America to Africa. Data shows that in the UK, one-third of healthcare workers had faced violence at the workplace [3]. In India, almost 75% of the doctors had dealt with one or the other form of violence during their practice [4]. Nearly 50% of violence were reported in intensive care units (ICUs), and in 70% of the cases, the patient relatives were actively involved [4]. Israel, Bangladesh, and Pakistan had also reported similar incidents [5-8]. The flawed healthcare system had led to a deteriorating relationship between the patient & doctor in China as well [9]. The Chinese Medical Doctor Association had reported more than 105 violent incidents between 2009 and 2015, where doctors were badly injured [10]. More than 100 healthcare workers died due to violence in the USA, between 1980 and 1990 [11]. As per the Indian Medical Association, over 80% of doctors are stressed out in their profession and nearly 56% don't sleep comfortably for 7 hours a day (Figure 1).

**Figure 2:**
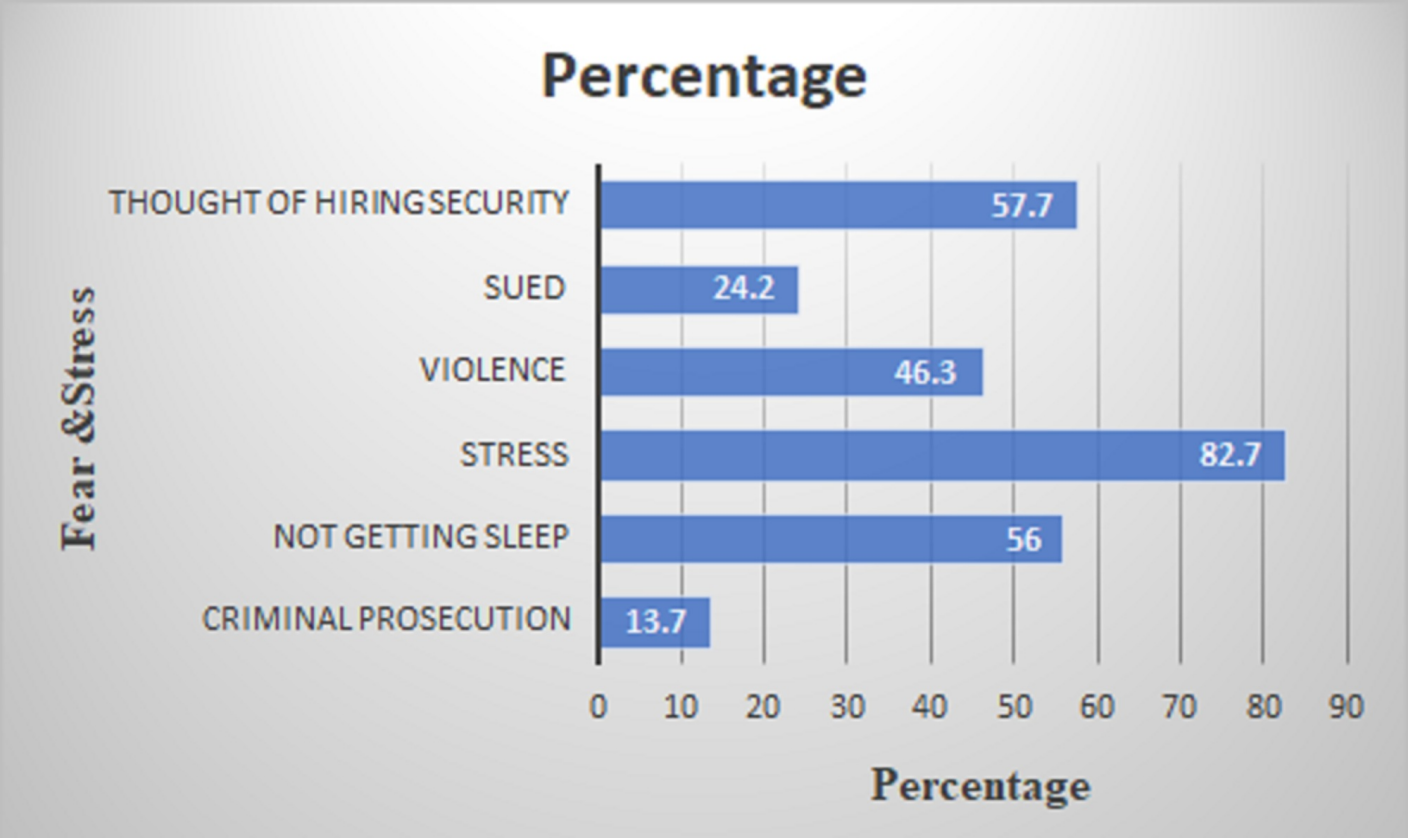
Fear and stress factors among Indian doctors

The same Indian Medical Association report states that 46% feel violence is the main cause of stress (Figure 2).

Timeline of major recent incidents in India

31 October 2019: Two residents of Banaras Hindu University, were beaten by patient attendants and goons in the parking space who escaped after the assault on motorbikes in Varanasi [12].

14 June 2019: A doctor was tied to a tree, robbed of his money and belongings; his wife and daughter were gang-raped in the Gaya district of Bihar [13].

11 June 2019: A junior resident doctor at Nil Ratan Sircar Medical College Hospital was hurled a brick to the head by the relatives of a 75-year-old patient. He suffered from a skull fracture and needed ICU admission [14].

5 June 2019: A Kolkata doctor was manhandled by the mob after the death of a six years old child in the Garden Reach area [15].

21 May 2019: The drunk attendants of a patient assaulted the Nizam's Institute of Medical Sciences (NIMS) doctor, Telangana [16].

12 May 2019: The medical officer in Dikom tea garden suffers from fractured ribs and broken bones after being assaulted by a mob in Assam [17].

27 February 2019: A junior doctor in the pediatric department of Gandhi Hospital, Hyderabad was assaulted by the grandmother of a 2-month-old baby who succumbed during treatment [18].

1 January 2019: A senior pediatrician was brutally assaulted by the patient’s family at his clinic in Himayatnagar Hyderabad, Telangana [19].

9 February 2019: An on-duty ophthalmologist at the Central Hospital North Eastern Coalfields (NEC) Coal India LTD, Digboi, Assam was assaulted by some miscreants [20].

23 December 2018: A doctor on duty in the ICU of Kolkata Nursing Home was harassed and physically assaulted by the relatives of a patient complaining of chest pain [21].

2 December 2018: A doctor was assaulted by the villagers at the District Headquarter Hospital, Bhadrak, Odisha after the refusal of Mahaprayan Vehicle [22].

25 November 2018: Two nursing staff were physically assaulted by the patient attendants on night duty at the Patna Medical College and Hospital (PMCH) [23].

November 2018: A trainee doctor at the Chengelpettu Medical College, Tamil Nadu was assaulted by inebriated patient attendants [24].

15 October 2018: The relatives of a patient assaulted a reserved category doctor demanding the service of an upper-class doctor at Jabalpur, Madhya Pradesh [25].

30 August 2018: A police officer, who underwent wrist surgery, slapped the Junior doctor without provocation at the Calcutta Medical Research Institute (CMRI) hospital, Ekbalpore, West Bengal [26].

19 May 2018: A patient's relative beat up two resident doctors of JJ Hospital, Mumbai [27].

15 March 2017: An orthopedician at the Dhule government Hospital, Maharashtra, who was brutally assaulted by a patient's kin develops blurring of vision in one eye [28].

August 2016: The relatives of an alcoholic deceased patient brutally thrashed two medical residents working at the Sassoon Medical College [29].

## Review

Causes of violence

**Figure 3:**
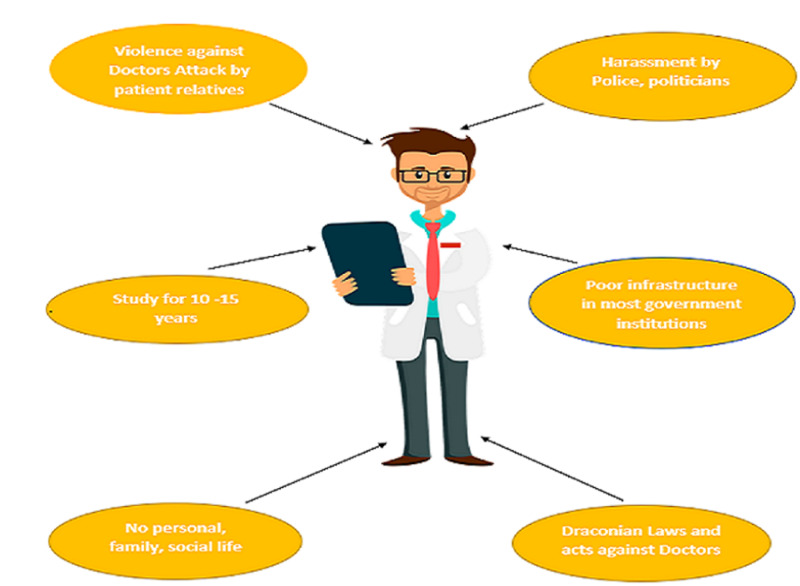
Factors stressing Indian doctors

In India, more than 80% of doctors are stressed out due to various reasons like violence against them, harassment by police as well as politicians, long duration of the study, lack of personal or social life, etc as illustrated in Figure 3.

The setting of violence against healthcare workers in India is different from that in the West. In India, violence perpetrators are mainly patient relatives, unknown sympathizers, criminal offenders, and even politicians [30]. In India, hardly 33% of the healthcare expenditure is borne by the government, the rest by the patients themselves. Low insurance penetration is another factor [31-32]. Unexpected healthcare expenses often push families into a trap of debt and financial instability. Here, within the background of smoldering anxiety of financial implications, verbal abuses easily escalate to violence. However, in the western world, the majority of the incidents occur during the night, in intensive care units and psychiatric and pediatric wards. Financial anxiety is not a causal factor in these countries as the healthcare expenditure is borne by the government [33]. The main offenders are the patients under the influence of drugs, alcohol, or psychiatric patients or their close relatives [34-35].

The causes of violence can be broadly categorized as poor quality healthcare in the majority of govt sector, the negative image of doctors and the role played by media, poor socioeconomic status of the patient and the ever-rising cost of the treatment, poor communication, vulnerability and susceptibility of hospitals, low awareness and knowledge about health-related issues, lax security or Inadequate security arrangement, mob mentality, and instant justice.

A study by Verma et al. showed that young doctors are more prone and female doctors face more violence [36]. The highest rates of violence were reported in the Obstetrics and Gynaecology department, followed by the Department of Internal Medicine and Surgery. The same study also mentioned that the most common causes of violence are longer waiting periods, delay in attending the patient, and admission denial.

Most commonly, workplace violence against doctors is seen in the Casualty or Emergency department followed by other departments. Almost all doctors had reported verbal violence in the emergency or casualty department. Invariably the resident doctors are the first target of the irate mob. Male doctors face workplace violence more often than females.

**Figure 4:**
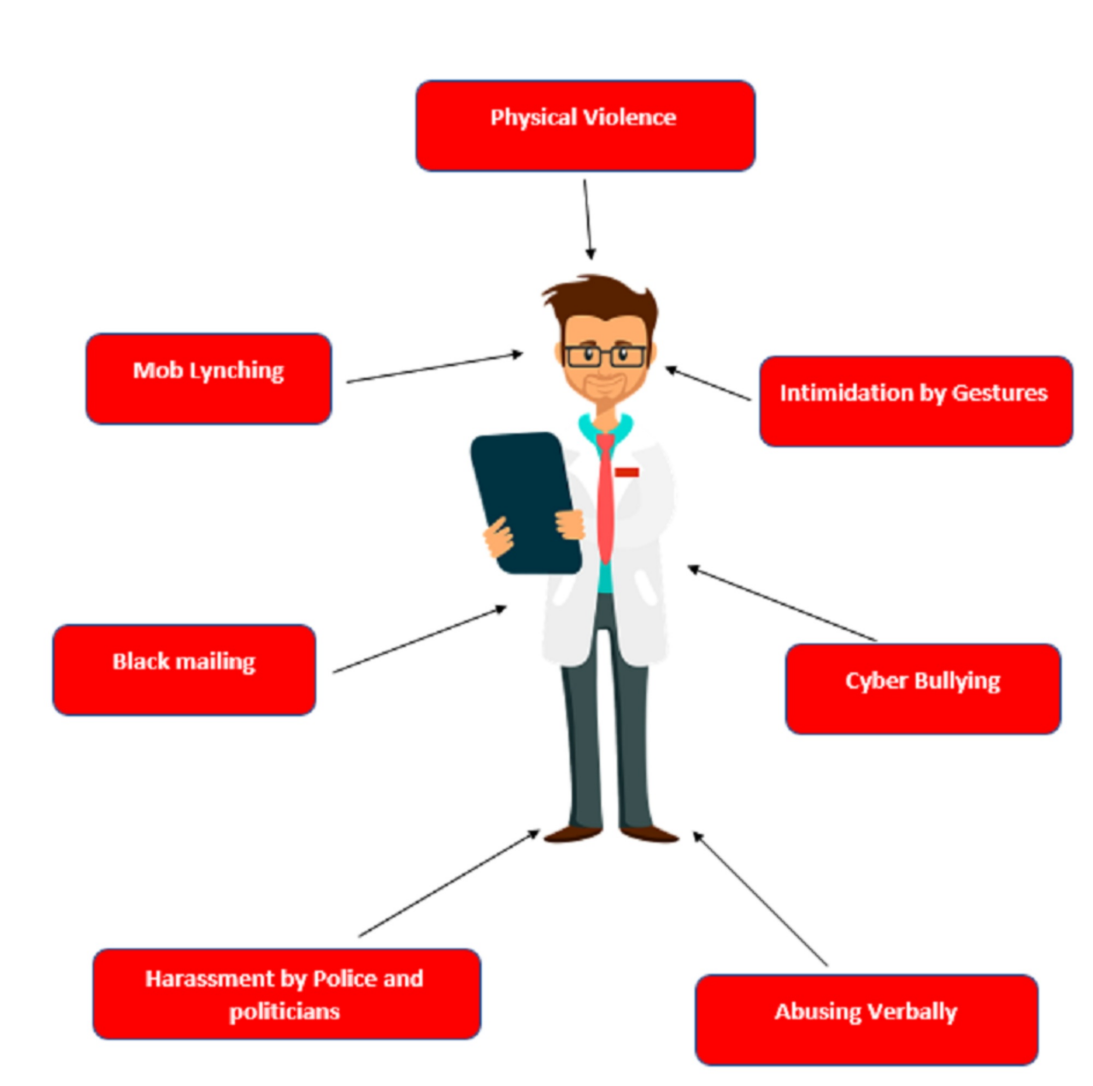
Most common types of violence against Indian doctors

Violence against doctors can be of any form like verbal abuse, physical violence, mob lynching harassment, etc. as can be seen in Figure 4. Verbal violence was the foremost common sort of violence.

**Figure 5:**
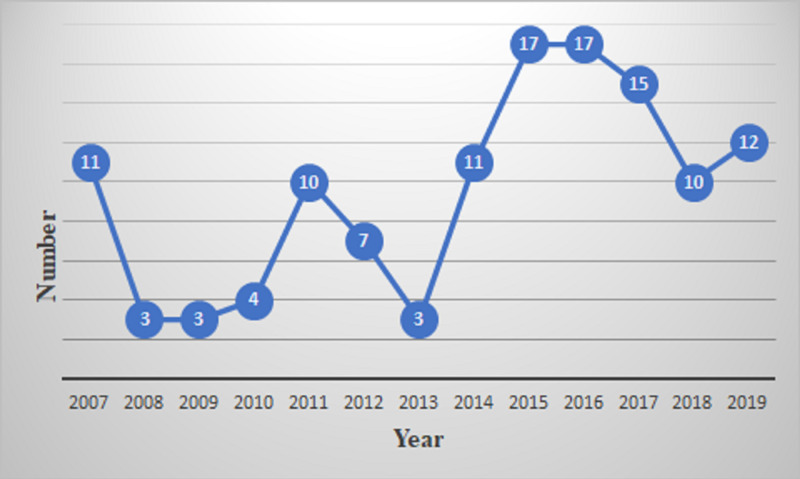
Violence against doctors leading to the number of strikes in hospitals reported in India from January 2007 to November 2019

Violence against doctors had invariably resulted in a strike in the hospital over the years as cited in Figure 5.

Warning signs of violence

The STAMP (Staring, Tone, Anxiety, Mumbling, Pacing) approach alerts the physician by looking for early warning signs of violence. The STAMP approach consists of the following [37]:

Staring is a prospective sign of violence. Staring was to threaten them into a quicker response.

The tone and volume of voice are connected with violent episodes. Most cases involve not only raised voices and yelling but also sarcasm and caustic replies.

Anxiety in coming to the emergency department makes patients stressed out. Before it reaches dangerous levels, ideally the doctor intervenes, but sometimes, it escalates to violence due to patient anxiety.

Mumbling is an indication of violence as it reflects frustration. Pacing by relatives is seen as a symbol of agitation.

Prevention of violence against doctors

**Figure 6:**
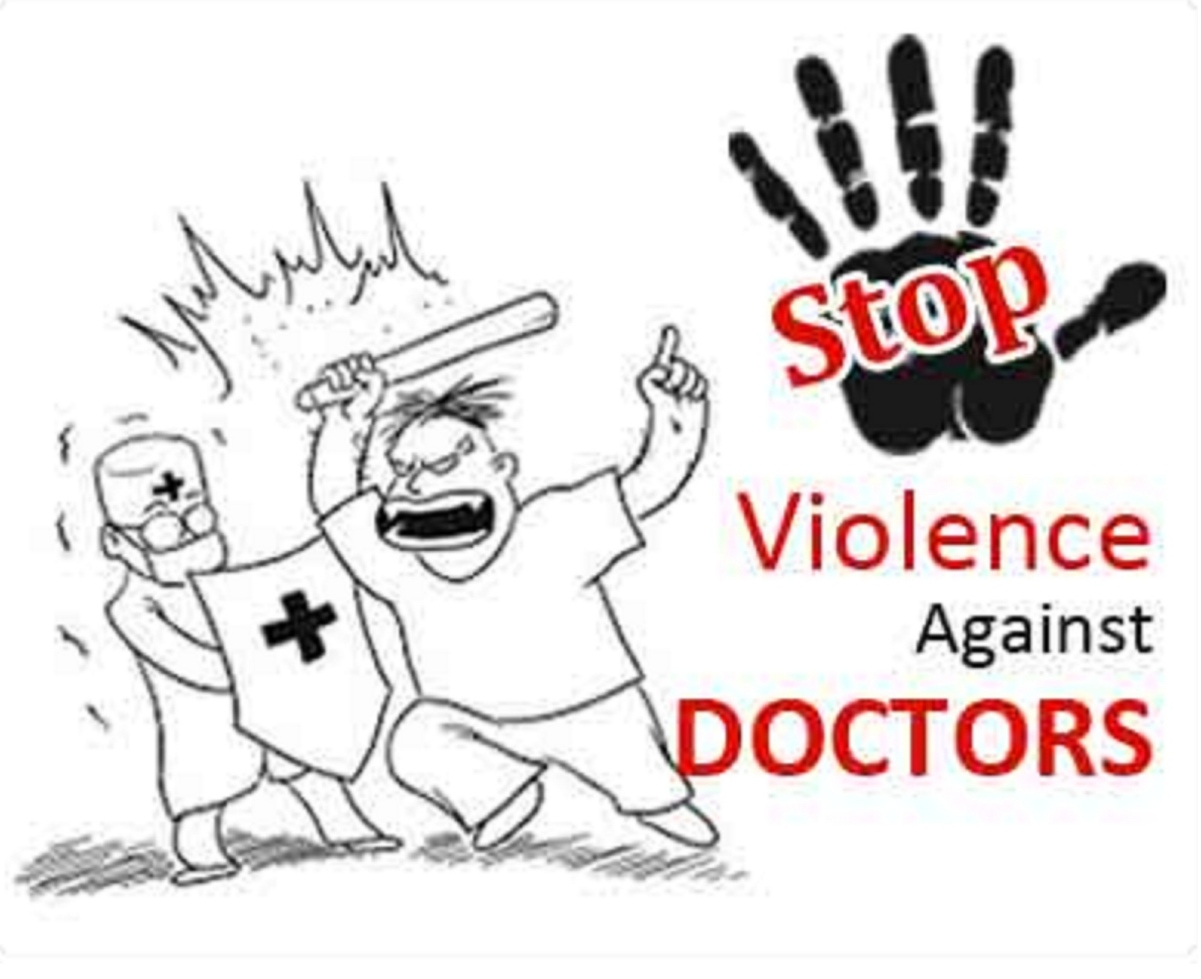
Stop violence against doctors (Retrieved from https://medicaldialogues.in/how-to-reduce-violence-against-doctors)

With the rise in mob violence incidents, doctors are also becoming cautious and reluctant to take up serious and grave cases, which in turn compromises healthcare which defeats the purpose of the healthcare delivery system. Unless we make the hospital environment free from fear and violence, the doctors won’t be able to work to their maximum potential, and hence, stopping the violence against doctors is of paramount importance (Figure 6). 

Several steps can be taken in this direction. They are as follows:

Government Policies and Responsibilities

Change in government policies like increased government spending on healthcare, improving the infrastructure of the hospitals, stricter implementation of rules, laws, and punishment for violence under the Prevention of Violence against Doctors and Hospitals according to appropriate Acts and relevant sections of the Indian Penal Code (IPC), violence against health-care personnel and hospitals should be made a nonbailable offense and damages should be recovered from the persons responsible for the violence. The government should take responsibility for the safety of healthcare workers.

Steps Doctor Should Take

The doctors should take a few precautions while taking valid and informed consent. Audio-visual consent is preferred. Proper documentation has to be done. The next important thing is communication which should be done preferably in the patient’s native language. Improving communication skills is another aspect of it. They shouldn't try to overdo or overreact and remain calm and composed.

Steps Institution Should Take

The standard operating procedure should be made and followed strictly. Code Purple should be declared and all measures should be taken in case of violence. Security staff to respond and assist immediately. All staff (except Operation Theatre & ICU) should form a human chain. All staff should remain calm & practice restraint. Closed-circuit television (CCTV) monitoring in sensitive areas is a must and should report to the Police immediately

Steps to be Taken by The Patient

Be aware of the health situation. Doctors practice medicine they can’t do magic and certainly, not everyone can be revived. Doctors cannot be held responsible for every death occurring in the hospital on the ground of negligence. Cost increases with the type of treatment & its advancement. If not satisfied with a doctor can speak to the concerned authority and take an appropriate decision to continue treatment or not.

Steps to be Taken by Media

Must put forward the unbiased news. It shouldn’t sensitize the news. It should highlight the doctor's predicament and the causes for the rise in violence against them.

Role of Medical Schools

The medical school can play an important role in creating awareness about workplace violence against doctors. Along with the medical subjects they should also teach about patient-doctor relations, communication with the patient in an effective way, empathy towards the patients and their relatives. The school should also teach them how to handle tactfully when the patients or their relatives behave aggressively and the situation turns chaotic and violent. They should teach them how to remain calm and responsible during those times without compromising the quality of patient care as well as safety.

The above steps can prevent workplace violence against doctors but the most important thing among them is communication by the doctor and the hospital. Proper communication can allay the situation or defuse the crisis. A senior doctor or someone from the management (preferably a senior one) should be able to communicate the condition and seriousness of the injury/disease of the patient to the relatives or attenders. A calm composed doctor with good communication skills can avert the situation most of the time. Along with that, the hospital security staff has to take steps to diffuse the mob and prevent violence against doctors and these hospitals. Police should be informed and kept vigilant if there is any suspicion of potential mob violence.

On the other way, the government should make stringent laws for violence against doctors in the workplace and punish the culprits. The government should improve the infrastructure of the hospitals and spend at least 5% GDP on healthcare which is neglected a lot. The media should avoid sensationalizing the news and put forward the unbiased news. Last but not the least patient education and creating awareness is a very important step towards preventing workplace violence against doctors.

## Conclusions

Unless there is an entire overhaul of the prevailing healthcare system, it is a herculean task to curb violence against doctors. The medical curriculum should include soft skills and communication skills required to empathize, remain calm, and twiddling my thumbs regardless of repeated prodding by the anxious patients. Along with this increased communication between the doctors and patients, filling crucial gaps in communication between doctors, patients, and relatives will help in mitigating the violence in a long way.
